# ABCD3-I score and the risk of early or 3-month stroke recurrence in tissue- and time-based definitions of TIA and minor stroke

**DOI:** 10.1007/s00415-017-8720-8

**Published:** 2018-01-11

**Authors:** Lukas Mayer, Julia Ferrari, Stefan Krebs, Christian Boehme, Thomas Toell, Benjamin Matosevic, Alexander Tinchon, Michael Brainin, Thomas Gattringer, Peter Sommer, Peter Thun, Johann Willeit, Wilfried Lang, Stefan Kiechl, Michael Knoflach, For the Austrian Stroke Unit Collaborators

**Affiliations:** 1 Department of Neurology, Medical University of Innsbruck, Anichstraße 35, 6020 Innsbruck, Austria; 2 Department of Neurology, Hospital Barmherzige Brueder, Vienna, Austria; 3 Department of Neurology, Landeskrankenhaus Feldkirch, Feldkirch, Austria; 4 Department of Neurology, University Clinic St. Pölten, Sankt Pölten, Austria; 5 Department of Clinical Neurosciences and Preventive Medicine, Danube University Krems, Krems an der Donau, Austria; 6 Department of Neurology, Medical University of Graz, Graz, Austria; 7 Department of Neurology, Hospital Rudolfstiftung Wien, Vienna, Austria; 8 Department of Neurology, Hospital Wilhelminenspital Wien, Vienna, Austria

**Keywords:** Transient ischemic attack, Minor stroke, ABCD3-I, Risk prediction, Time-based, Tissue-based

## Abstract

Changing definition of TIA from time to a tissue basis questions the validity of the well-established ABCD3-I risk score for recurrent ischemic cerebrovascular events. We analyzed patients with ischemic stroke with mild neurological symptoms arriving < 24 h after symptom onset in a phase where it is unclear, if the event turns out to be a TIA or minor stroke, in the prospective multi-center Austrian Stroke Unit Registry. Patients were retrospectively categorized according to a time-based (symptom duration below/above 24 h) and tissue-based (without/with corresponding brain lesion on CT or MRI) definition of TIA or minor stroke. Outcome parameters were early stroke during stroke unit stay and 3-month ischemic stroke. Of the 5237 TIA and minor stroke patients with prospectively documented ABCD3-I score, 2755 (52.6%) had a TIA by the time-based and 2183 (41.7%) by the tissue-based definition. Of the 2457 (46.9%) patients with complete 3-month followup, corresponding numbers were 1195 (48.3%) for the time- and 971 (39.5%) for the tissue-based definition of TIA. Early and 3-month ischemic stroke occurred in 1.1 and 2.5% of time-based TIA, 3.8 and 5.9% of time-based minor stroke, 1.2 and 2.3% of tissue-based TIA as well as in 3.1 and 5.5% of tissue-based minor stroke patients. Irrespective of the definition of TIA and minor stroke, the risk of early and 3-month ischemic stroke steadily increased with increasing ABCD3-I score points. The ABCD3-I score performs equally in TIA patients in tissue- as well as time-based definition and the same is true for minor stroke patients.

## Introduction

TIA and minor stroke are both conditions with high risk for early neurologic worsening and recurrent ischemic stroke [1]. To estimate the individual risk, scores have been proposed with the ABCD2 and ABCD3-I (Table 1) scores being the best validated and predominantly used risk evaluators in TIA patients [2–4]. The original definition of a TIA in the late 1950s was time-based relying mainly on symptom duration < 24 h [5–8]. Advances in and availability of cerebral imaging since then have led to the suggestion of a re-definition of TIA based on imaging findings depending on the absence of an ischemic brain damage [9]. The tissue-based approach may assist in differentiating TIA from minor stroke patients earlier than the time-based definition as patients who are admitted earlier than the 24-h time window for symptom relief could not be divided between the two. This imaging-based definition is highly likely to be implemented in the upcoming International Classification of Diseases (ICD) 11 [10]. The ABCD2 score has previously been shown to work equally in the tissue-based definition of TIA and minor stroke [11]. The aim of this study was to evaluate the prognostic value of the more advanced ABCD3-I score in tissue- as well as time-based definitions of TIA and minor stroke.

**Table 1:** ABCD^3^- and ABCD^3^-I-score

Score variables	ABCD^3^-score	ABCD^3^-I-score
Age ≥ 60 years	1	1
Blood pressure ≥ 140/90 mmHg	1	1
Clinical features of TIA		
Speech impairment only	1	1
Unilateral weakness	2	2
Duration of TIA		
10–59 min	1	1
≥ 60 min	2	2
Diabetes mellitus present	1	1
Dual TIA^a^	2	2
Imaging criteria		
Ipsilateral ≥ 50% ICA stenosis	–	2
Acute diffusion-weighted imaging hyperintensity	–	2
Total range of score points	0–9	0–13

^a^TIA prompting medical attention plus at least one other TIA in the preceding 7 days

## Materials and methods

Data were collected from the Austrian Stroke Unit Registry which is prospectively filled with data from 37 stroke units all over Austria. This registry was established in 2003 and documents patient characteristics, diagnostic and clinical information upon admission, discharge and 3-month followup on every person who was admitted to a stroke unit in Austria. High quality data are generated through immediate documentation, online plausibility checks and standardized variable definitions. A registry-expansion to include various TIA and minor stroke-related variables was done between December 2010 and January 2014.

The study population consists of adult patients suffering TIA or minor stroke (defined as a score of 4 or less on the NIHSS), who were admitted within the first 24 h after symptom onset, had an in-hospital delay (defined as duration between hospital and stroke unit admission) of less than 6 h and a full documentation for ABCD3-I-score. The diagnosis of TIA or minor stroke was done by the treating stroke-specialist, who also decided on the appropriate diagnostic measures (MRI or CT scan). If the initial diagnosis of TIA or minor stroke turned out to be wrong during stroke unit stay, the patient was classified as stroke mimic upon discharge and was not considered in the present analysis. Based on the symptom duration and the presence or absence of a DWI lesion on MRI or a corresponding lesion on CT, TIA or minor stroke were categorized into four different groups: (1) time-based TIA (symptom duration < 24 h, irrespective of imaging findings) or (2) time-based minor stroke (symptom duration ≥ 24 h, no consideration of neuroimaging information) as well as (3) tissue-based TIA (no ischemic infarct on brain imaging) or (4) minor stroke (brain MRI or CT depict an ischemic lesion). ABCD3-I score was defined and calculated as described previously [3, 12]. In the ABCD3-I score, symptom duration above 60 min as well as a brain lesion in brain CT or MRI score 2 points each. Therefore, the minimum ABCD3-I score is 2 in the time- as well as the tissue-based definition of minor stroke, and a maximum ABCD3-I score of 11 is possible in the tissue-based TIA definition.

Early stroke was defined as recurrent or progressive ischemic stroke during stroke unit stay associated with neurological worsening of more than one point on the NIHSS score. Stroke recurrence after discharge was documented in a followup assessment either by phone or in person 90 ± 14 days after the index event. “3-month stroke” was defined as stroke during stroke unit stay or within the 90 ± 14 day followup period.

### Statistical analysis

Data processing was done through R, version 2.15.2 (R Foundation for Statistical Computing, Vienna, Austria). The performance of the ABCD3-I score in different definitions of TIA and minor stroke was done by receiver operating characteristic (ROC) curves.

### Standard protocol approvals, registrations, and patient consents

This registry represents a quality of stroke unit care assessment measure founded on the Austrian federal law promoting quality of health financed by the Austrian Federal Ministry of Health. Data are being collected in an anonymized fashion. Further details concerning the registry have already been published [12]. The current analysis has been approved by an institutional review board.

## Results

The Austrian Stroke Unit Registry documented 35,853 patients between December 2010 and January 2014, 14,862 of which had a TIA or minor stroke. After excluding patients younger than 18 years of age, not admitted within 24 h after symptom onset or with an in-hospital delay of more than 6 h, with no information on the neurological deficit at discharge from the stroke unit and those who received endovascular treatment, 10,307 remained. In 5237 of these patients, ABCD3-I values were fully documented and, therefore, study inclusion and further analysis were possible. 3-month followup data were available for 2457 patients. No relevant differences were seen in patients with and without full dataset [12].

Using a time-based definition, 2755 (52.6%) patients had a TIA and 2482 (47.4%) had a minor stroke. Respectively, 1195 (43.4%) and 1262 (50.8%) of these subjects had complete 3-month followup data. On the basis of a tissue-based definition, 2183 (41.7%) had a TIA and 3054 (58.3%) had a minor stroke. Complete followup data were available for 971 (44.5%) and 1486 (48.7%) of these subjects, respectively.

Early stroke could be seen in 1.1% (29 of 2755 patients) of time-based TIA, 3.8% (94 of 2482 patients) of time-based minor stroke, 1.2% (27 of 2183 patients) of tissue-based TIA and 3.1% (96 of 3054 patients) of tissue-based minor stroke patients. Of those with complete followup data, 3-month stroke was recorded in 2.5% (30 of 1195 patients) of time-based TIA, 5.9% (74 of 1262 patients) of time-based minor stroke, 2.4% (23 of 971 patients) of tissue-based TIA and 5.5% (81 of 1486 patients) of tissue-based minor stroke.

Characteristics and demographical data comparing all four groups can be found in Table 2. Vascular risk conditions, as well as atrial fibrillation were more frequent in minor stroke than in TIA patients irrespective of the definition used. The groups did not differ relevantly in the duration of the stroke unit stay. All patients with TIA or minor stroke received brain imaging prior to or during stroke unit stay. Early treatment within the first 2 days included platelet inhibition, low-dose heparin and medium-to-high dose heparin and were similarly distributed in both definitions of TIA and minor stroke. Frequency of imaging modality and early treatment in all subgroups can be seen in Table 2.

**Table 2:** Characteristics of the whole study population and of subgroups dependent on time- or tissue-based definition of TIA and minor stroke (MS)

	All	Time-based TIA	Time-based MS	Tissue-based TIA	Tissue-based MS
Number of patients	5237	2755	2482	2183	3054
Age, median (Q1–Q3)	71.9 (61.6–80.2)	72.1 (61.4, 80.4)	71.6 (61.9, 79.8)	70.9 (59, 79.7)	72.4 (63.2, 80.4)
Male sex, % (*n*)	55.7 (2915)	53.5 (1473)	58.1 (1442)	52.9 (1154)	57.7 (1761)
Hypertension, % (*n*)	78.5 (4113)	76.8 (2116)	80.5 (1997)	72.9 (1592)	82.5 (2521)
Diabetes mellitus, % (*n*)	23 (1203)	21.9 (602)	24.2 (601)	19.7 (430)	25.3 (773)
Hypercholesterolemia, % (*n*)	61.4 (3214)	60.8 (1676)	62 (1538)	58.1 (1268)	63.7 (1946)
Atrial fibrillation, % (*n*)	19.3 (1010)	17.7 (487)	21.1 (523)	15.9 (348)	21.7 (662)
Current smoking, % (*n*)	19.5 (1021)	17.7 (488)	21.5 (533)	17.5 (382)	20.9 (639)
Pre stroke mRS 0–2, % (*n*)	92.3 (4828)	91.7 (2522)	93 (2306)	93.7 (2042)	91.3 (2786)
NIHSS upon admission, median (Q1–Q3)	1 (0, 2)	0 (0, 2)	2 (1, 3)	1 (0, 2)	2 (0, 2)
Onset to door time, median (Q1*–*Q3)	120 (68, 255)	108 (60, 200)	151.5 (80, 345)	119 (60, 230)	130 (70, 280)
Stroke unit stay in days, median (Q1–Q3)	2 (1, 3)	2 (1, 3)	2 (1, 4)	2 (1, 3)	2 (1, 4)
Imaging during stroke unit stay					
CT, %	86.6	86.6	86.7	85.6	87.4
MRI, %	71.0	70.4	63.2	59.4	79.3
Both, %	56.0	52.4	59.9	45.6	63.4
Early treatment					
Platelet inhibition, %	84.9	85.1	84.7	86.4	83.9
Low-dose heparin, %	75.9	75.7	76.2	76.9	75.2
Medium-to-high dose heparin, %	15.3	13.0	17.7	12.4	17.2
Secondary prevention					
Statins, %	68.0	66.5	69.2	63.5	71.2
Antihypertensive medication, %	77.0	76.0	78.2	71.5	80.9
Early stroke, % (*n*)	2.3 (123)	1.1 (29)	3.8 (94)	1.2 (27)	3.1 (96)
Early stroke AUCs (95% CI)	0.664 (0.618–0.709)	0.645 (0.539–0.752)	0.605 (0.552–0.658)	0.651 (0.546–0.756)	0.598 (0.542–0.653)
3-month stroke, % (*n*)^a^	4.2 (104)	2.5 (30)	5.9 (74)	2.3 (23)	5.5 (81)
3-month stroke AUCs (95% CI)	0.646 (0.592–0.700)	0.588 (0.487–0.688)	0.637 (0.573–0.701)	0.561 (0.461–0.661)	0.619 (0.558–0.680)

^a^In those with available 3 month followup

**Fig. 1:**
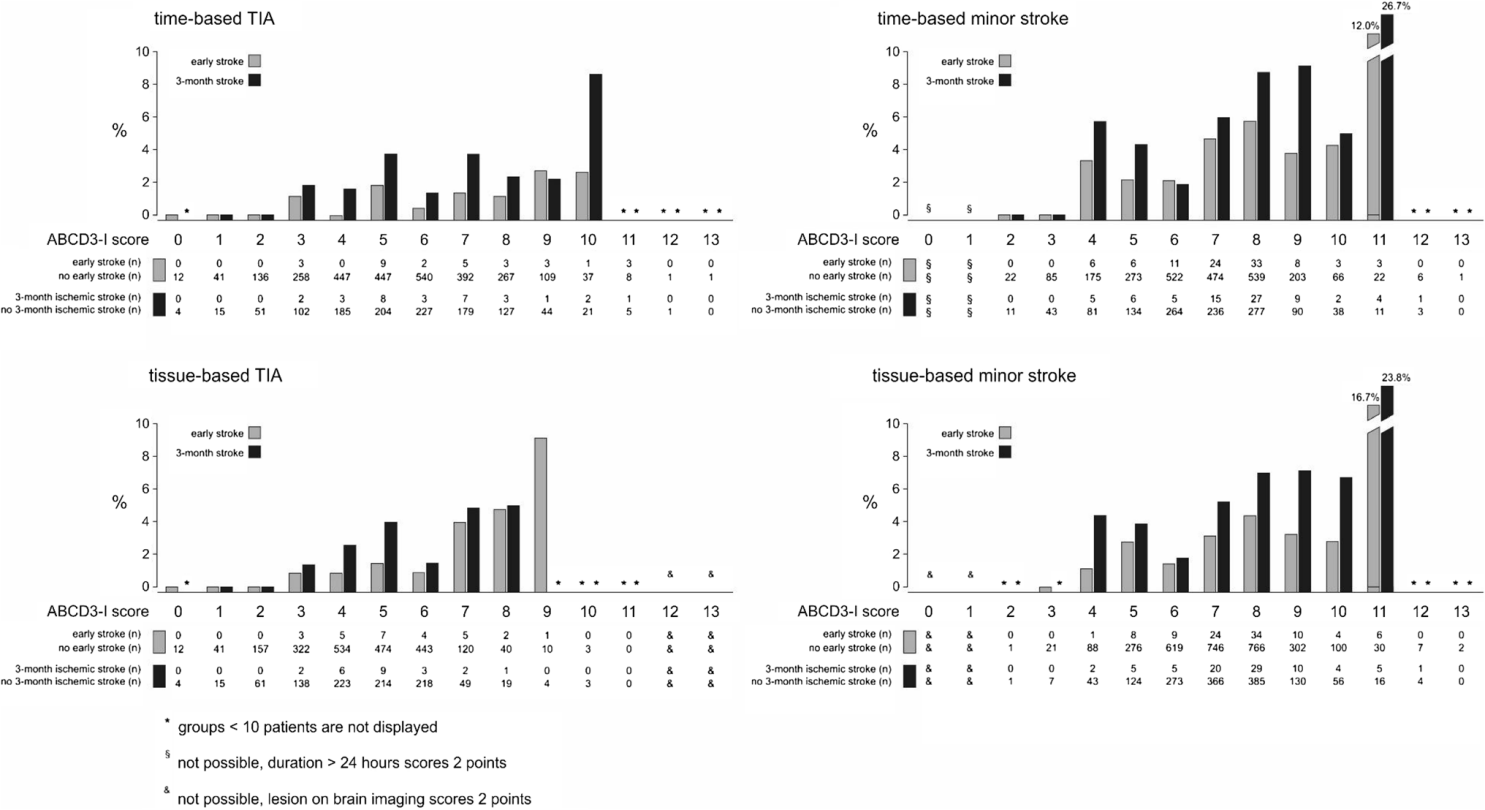
Risk of early or 3-month ischemic stroke dependent on ABCD3-I score points in time- and tissue-based definition of TIA and minor stroke

The risk of early stroke during stroke unit stay and 3-month stroke dependent on ABCD3-I scores are depicted in Fig. 1. In time- as well as tissue-based definitions of TIA and minor stroke, the risk of early or 3-month stroke increased with increasing score points (Fig. 1). Area under the ROC curve for ABCD3-I score has been calculated for the whole population and the different subgroups, and is listed in Table 2.

## Discussion

We present here the analysis of a large prospective cohort of patients admitted to an Austrian stroke unit with minor neurological symptoms within a median of 2 h after symptom onset (Table 2) in a phase when it is still unclear if the cerebral ischemia turns out to be a TIA or minor stroke. To the best of our knowledge, we are the first to demonstrate that increasing ABCD3-I score points are associated with a steady increase in early and 3-month stroke risk irrespective if TIA was later classified according to the classic time-based or the upcoming tissue-based definition. The same was true for those patients later classified as minor stroke on a time or tissue-defined basis. The prognostic performance of the ABCD3-I score as measured by the area under the ROC curve was similar in all definitions of TIA and minor stroke.

This finding harbors two important consequences: first, it supports the common trend to select TIA patients with a high risk for recurrent or progressive stroke very early after symptom onset based on TIA risk scores for an intensified antithrombotic treatment (as done in the CHANCE and SOCRATES trials [13, 14]). Second, the increase in risk is similarly independent of the definition of TIA applied and extends to patients later classified as minor stroke.

The strength of our study lies in the large and prospectively collected multi-center cohort receiving early and intensive stroke unit care. Additionally, diagnosis, treatment and outcome assessment were performed by stroke specialists only making the inclusion of TIA/stroke mimics improbable. A relevant selection bias due to stroke unit admission or incomplete dataset is unlikely as the distribution of patients characteristics was similar in those with and without complete information on the ABCD3-I score, with and without 3-month followup and did equal to those of other large observational TIA cohorts, as previously summarized in 12. Still our results have to be interpreted with caution: our pre-specified early stroke endpoint summarizes recurrent and progressive stroke, two entities with potentially different pathophysiology and 3-month followup was available only in about half of the patients. Furthermore, MRI was less frequently performed in the group with tissue-based TIA compared to those with tissue-based minor stroke. We cannot exclude that patients in the tissue-based TIA group with CT imaging only might have shown lesions in MRI. Still—as the ABCD3-I score performs similar in both groups—this imbalance is unlikely to change our results.

In conclusion, the ABCD3-I score works in both—TIA and minor stroke patients—irrespective of which definition is applied.

## Abbreviation

TIA: Transient ischemic attack

## References

[1] Coull AJ, Lovett JK, Rothwell PM, Oxford Vascular Study (2004). Population based study of early risk of stroke after transient ischaemic attack or minor stroke: implications for public education and organisation of services. BMJ.

[2] Johnston SC, Rothwell PM, Nguyen-Huynh MN, Giles MF, Elkins JS, Bernstein AL (2007). Validation and refinement of scores to predict very early stroke risk after transient ischaemic attack. Lancet Lond Engl.

[3] Merwick A, Albers GW, Amarenco P, Arsava EM, Ay H, Calvet D (2010). Addition of brain and carotid imaging to the ABCD^2^ score to identify patients at early risk of stroke after transient ischaemic attack: a multicentre observational study. Lancet Neurol.

[4] Song B, Fang H, Zhao L, Gao Y, Tan S, Lu J (2013). Validation of the ABCD3-I score to predict stroke risk after transient ischemic attack. Stroke.

[5] Fisher C, Wright IS, Millikan CH (1958). Intermittent cerebral ischemia. Transactions of the second conference held under the auspices of the American Heart Association.

[6] Acheson J, Hutchinson EC (1964). Observations on the natural history of transient cerebral ischaemia. Lancet Lond Engl.

[7] Marshall J (1964). The natural history of transient ischaemic cerebro-vascular attacks. Q J Med.

[8] (1975) A classification and outline of cerebrovascular diseases. II. Stroke 6(5):564–616 10.1161/01.str.6.5.564 1179466

[9] Easton JD, Saver JL, Albers GW, Alberts MJ, Chaturvedi S, Feldmann E (2009). Definition and evaluation of transient ischemic attack: a scientific statement for healthcare professionals from the American Heart Association/American Stroke Association Stroke Council; Council on Cardiovascular Surgery and Anesthesia; Council on Cardiovascular Radiology and Intervention; Council on Cardiovascular Nursing; and the Interdisciplinary Council on Peripheral Vascular Disease. The American Academy of Neurology affirms the value of this statement as an educational tool for neurologists. Stroke.

[10] (2017) ICD-11 beta draft. http://apps.who.int/classifications/icd11/browse/f/en#/http%3a%2f%2fid.who.int%2ficd%2fentity%2f826335789. Accessed 4 Aug 2017

[11] Giles MF, Albers GW, Amarenco P, Arsava EM, Asimos AW, Ay H (2011). Early stroke risk and ABCD2 score performance in tissue- vs time-defined TIA: a multicenter study. Neurology.

[12] Knoflach M, Lang W, Seyfang L, Fertl E, Oberndorfer S, Daniel G (2016). Predictive value of ABCD2 and ABCD3-I scores in TIA and minor stroke in the stroke unit setting. Neurology.

[13] Wang Y, Johnston SC, CHANCE Investigators (2010). Rationale and design of a randomized, double-blind trial comparing the effects of a 3-month clopidogrel-aspirin regimen versus aspirin alone for the treatment of high-risk patients with acute nondisabling cerebrovascular event. Am Heart J.

[14] Johnston SC, Amarenco P, Albers GW, Denison H, Easton JD, Held P (2015). Acute stroke or transient ischemic attack treated with aspirin or ticagrelor and patient outcomes (SOCRATES) trial: rationale and design. Int J Stroke Off J Int Stroke Soc.

